# Applying an internal transcribed spacer as a single molecular marker to differentiate between *Tetraselmis* and *Chlorella* species

**DOI:** 10.3389/fmicb.2023.1228869

**Published:** 2023-08-23

**Authors:** Wael A. Fathy, Natascha Techen, Khaled N. M. Elsayed, Ehab A. Essawy, Eman Tawfik, Khairiah Mubarak Alwutayd, Mohamed S. Abdelhameed, Ola Hammouda, Samir A. Ross

**Affiliations:** ^1^Department of Botany and Microbiology, Faculty of Science, Beni-Suef University, Beni-Suef, Egypt; ^2^Faculty of Science and Informatics, Doctoral School of Biology, University of Szeged, Szeged, Hungary; ^3^National Centre for Natural Products Research, Research Institute of Pharmaceutical Sciences, School of Pharmacy, University of Mississippi, Oxford, MS, United States; ^4^Biochemistry Division, Department of Chemistry, Faculty of Science, Helwan University, Helwan, Egypt; ^5^Department of Botany and Microbiology, Faculty of Science, Helwan University, Helwan, Egypt; ^6^Department of Biology, College of Science, Princess Nourah bint Abdulrahman University, Riyadh, Saudi Arabia; ^7^Division of Pharmacognosy, Department of BioMolecular Sciences, School of Pharmacy, University of Mississippi, Oxford, MS, United States

**Keywords:** chlorophyta, DNA barcoding, microalgae identification, internal transcribed spacer (ITS), *Tetraselmis* sp., *Chlorella* sp.

## Abstract

In the realm of applied phycology, algal physiology, and biochemistry publications, the absence of proper identification and documentation of microalgae is a common concern. This poses a significant challenge for non-specialists who struggle to identify numerous eukaryotic microalgae. However, a promising solution lies in employing an appropriate DNA barcoding technique and establishing comprehensive databases of reference sequences. To address this issue, we conducted a study focusing on the molecular characterization and strain identification of *Tetraselmis* and *Chlorella* species, utilizing the internal transcribed spacer (ITS) barcode approach. By analyzing the full nuclear ITS region through the Sanger sequencing approach, we obtained ITS barcodes that were subsequently compared with other ITS sequences of various *Tetraselmis* and *Chlorella* species. To ensure the reliability of our identification procedure, we conducted a meticulous comparison of the DNA alignment, constructed a phylogenetic tree, and determined the percentage of identical nucleotides. The findings of our study reveal the significant value of the ITS genomic region as a tool for distinguishing and identifying morphologically similar chlorophyta. Moreover, our results demonstrate that both the ITS1 and ITS2 regions are capable of effectively discriminating isolates from one another; however, ITS2 is preferred due to its greater intraspecific variation. These results underscore the indispensability of employing ITS barcoding in microalgae identification, highlighting the limitations of relying solely on morphological characterization.

## Introduction

Chlorophyta are a diverse group of eukaryotic microorganisms encompassing approximately 8,000 identified species (Becker and Marin, 2009; Guiry, 2012). However, it is estimated that there are still ~5,000 undiscovered species, primarily in tropical and subtropical regions (Guiry, 2012). Within aquatic, freshwater, and terrestrial ecosystems, chlorophyta play a crucial role as primary producers (Torres et al., 2008). They also serve as valuable biological indicators for monitoring and preserving aquatic habitats (Omar, 2010). Additionally, chlorophyta offer a versatile platform for the synthesis of various bioproducts, including high-value bioactive and recombinant proteins; biofuels such as hydrogen and alcohols; isoprenoids; and nutritional supplements (Al-Haj et al., 2016; Fathy et al., 2021). Furthermore, they serve as appealing laboratory models for genetic research such as being selectable markers or producing specific pigments (Taparia et al., 2019; Cecchin et al., 2022). Chlorophyta exhibit significant ecological importance in both humid terrestrial and aquatic ecosystems, and their species are frequently employed as bioindicators in ecological studies and water monitoring (Torres et al., 2008; Hussein et al., 2022). Moreover, the utilization of green microalgae for biotechnological applications, including the production of fuels, chemicals, nanoparticles, foods, and animal feed, is gaining increasing popularity (Nascimento et al., 2013; Fathy et al., 2020).

Chlorophyta identification can be challenging, and it frequently requires a skilled expert to carefully examine live cultivated cells under a microscope (De Clerck et al., 2013). The existence of cryptic species and the phenotypic flexibility exhibited in some species, however, may prevent a clear morphological species diagnosis (Krienitz et al., 2015). Historically, the morphological characteristics of microalgae, including form, presence of chloroplast, pyrenoid location, and presence of flagella, have been used to classify them (Škaloud et al., 2005). General biological appearance and specific structure are examples of morphological features that can be also used to distinguish between species. In addition, features from physiology, such as those related to metabolism and secretions, and ecologies, such as those related to habitats, food, seasonal change, and biogeographic distribution, are used to identify species (Cunningham and Meghen, 2001; Moretti et al., 2003). The subjective interpretation of these characteristics as well as the possibility that some strains may lose crucial characteristics during prolonged laboratory cultivation, such as gas vesicles or colony form, could lead to mistaken identity (Gugger et al., 2002). Additionally, this method based on morphology needs specialized taxonomists and high-resolution equipment (Hall et al., 2010; Manoylov, 2014). Microalgal identification is considered a frequent issue mentioned in reports of microalgal biotechnological applications. Even if a strain came from a culture collection, it is possible that it was mislabeled when it was added to the collection or that it later became contaminated with another strain that has since outgrown the culture (Fawley and Fawley, 2020). The use of DNA barcodes and the creation of databases with reference sequences have been viewed as promising solutions for overcoming these challenges in microalgae identification (Alemzadeh et al., 2014).

DNA barcoding is a widely recognized technique for species identification that involves comparing DNA sequences of specimens with a reference database of predetermined species (Hebert et al., 2003). This effective approach has found applications in various fields such as taxonomy, ecology, biosecurity, and food product control (Carvalho et al., 2015). DNA barcoding is particularly valuable for identifying species with few or cryptic structural characteristics and for revealing cryptic diversity at different taxonomic levels, DNA-based identification is especially helpful (Costa et al., 2012; Pawlowski and Holzmann, 2014). It offers a reliable and rapid method for identifying green microalgae, regardless of their life stage (Buchheim et al., 2011).

**Figure 1:**
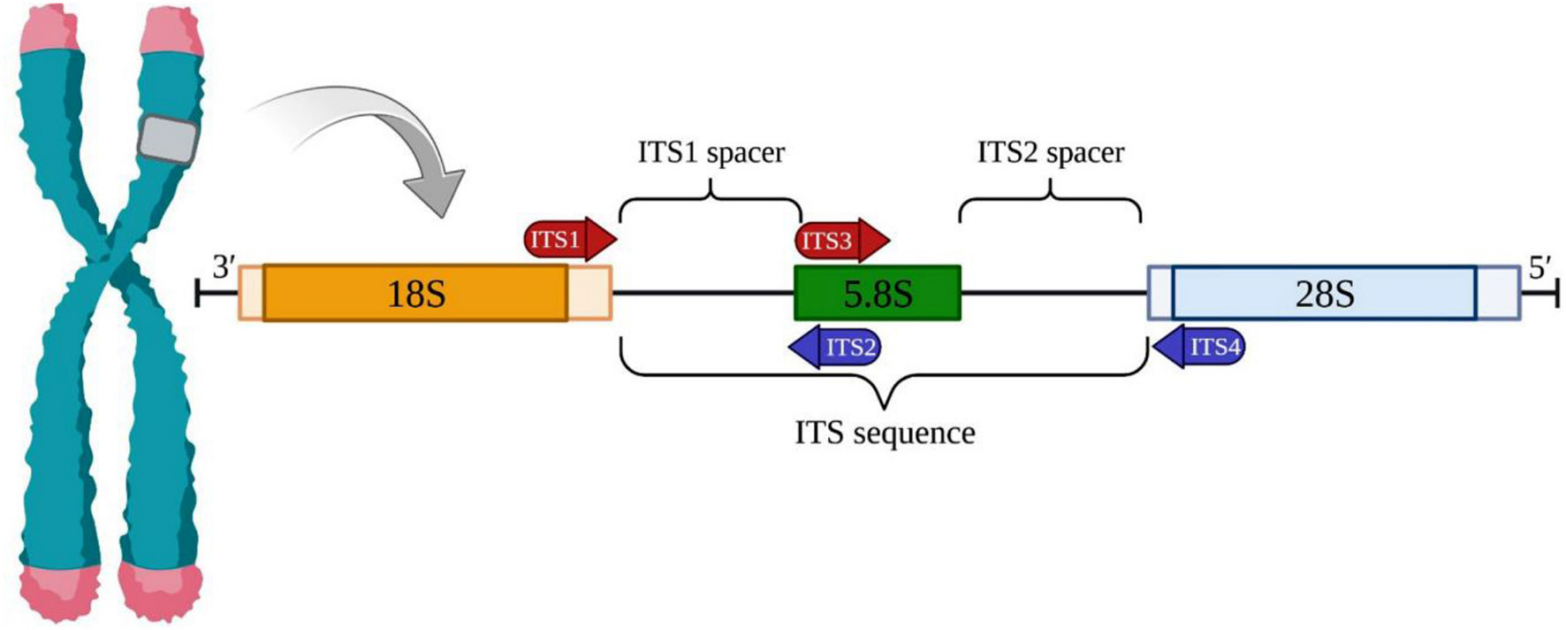
The nuclear ribosomal internal transcribed spacer (ITS) region. The ITS region in a eukaryotic cell is made up of two spacers, ITS1 and ITS2. ITS1 is located between the 18S and 5.8S rRNA genes, while ITS2 is located between the 5.8S and 28S rRNA genes. The entire ITS region can be amplified completely or just a portion by using universal forward and reverse primers.

The ITS1 and ITS2 markers are recognized as highly promising choices for microalgae barcoding (Caisová et al., 2011; Hegewald et al., 2013). These markers are derived from the fast-evolving genomic region located between the genes for ribosomal RNA. The ITS1-5.8S-ITS2 region can be amplified using a single pair of universal primers enabling the identification of nearly all photosynthetic species (Figure 1) (White et al., 1990). Although ITS1 and ITS2 are highly variable markers (Hall et al., 2010), it is feasible to investigate their primary sequences as well as their structural characteristics (Koetschan et al., 2010). By analyzing both nucleotide information and compensatory base fluctuations with secondary structure information, many of the limitations associated with this barcode can be overcome. Numerous taxonomic revisions in chlorophyta, particularly in taxa with straightforward morphology and limited ultrastructural features, have employed ITS1 and ITS2 as preferred molecular markers (Buchheim et al., 2011). The application of ITS1- and ITS2-based phylogenies has significantly altered the taxonomy of green microalgae (Krienitz et al., 2015). DNA barcoding for biological identification, first introduced by Hebert et al. (2003), relies on short, easily amplified DNA segments that exhibit high interspecies variability and minimal intraspecific variability (Stackebrandt and Goebel, 1994).

In this study, we sequenced the complete ITS regions of a *Chlorella* and *Tetraselmis* isolate to molecularly identify them. A comparative analysis of their sequences with other ITS sequences demonstrated the effectiveness of this genomic region as a reliable marker for distinguishing closely related species in the chlorophyta group. The inclusion of these two genera aimed to assess the universality of the ITS marker across a diverse range of microalgae, providing insights into its applicability. *Chlorella*, a well-studied representative of the chlorophyta phylum, was selected as a model alga due to its well-established characterization. *Tetraselmis*, a less-explored genus, offered valuable insights into the ITS marker's usability in a relatively unexplored microalgal group.

## Materials and methods

### Sampling and culturing medium

The microalgae species *Tetraselmis* and *Chlorella* were purchased from “Algae Research and Supply” (San Diego CA, USA). To confirm that the isolates were pure and axenic, they were morphologically examined using a microscope Nikon Eclipse 90i (Nikon Instruments Inc. Melville, NY USA) and cultured on both solid and liquid F/2 media.

### DNA isolation and ITS PCR protocol

A Retsch mixer mill MM200 (Retsch, Newtown, PA, USA) was used to grind 100 mg of fresh microalgae for 2 min. Corresponding to the manufacturer's recommendations, the DNA was extracted using a DNeasy Plant Mini Kit (Qiagen Inc., Valencia, CA, USA). The PCR was performed using a 25 μL reaction mixture that contained 2 μL of DNA, 2.5 μL 10x PCR reaction buffer, 0.5 μL of 10 mM dNTP mixture, 0.5 μL of each forward and reverse ITS primer (10 nmol each) (Table 1), 0.75 μL of 50 mM MgCl_2_, and 2 units of Platinum Taq DNA Polymerase 5 μ/μL (Invitrogen, Carlsbad, CA, USA). The PCR protocol was as follows: a 90 s initial denaturation step at 94°C, then 35 cycles for 30 s at 94°C, 20 s at 50°C, 60 s at 72°C, and a final extension for 90 s at 72°C. After amplification, the sample was analyzed on a 1% borate agarose gel, stained with ethidium bromide, and visualized using UV light. The PCR products were compared to the molecular size standard of 1 kb plus DNA ladder (Invitrogen, Carlsbad, CA). The electrophoresis was performed in a running buffer of 1% borax, with a voltage of 100 for a duration of 30 min.

**Table 1:** List of ITS and M13 primers used.

**Primer name**	**Sequence**	**Reference**
ITS1	TCCGTAGGTGAACCTGCGG	(White et al., 1990)
ITS4	TCCTCCGCTTATTGATATGC	(White et al., 1990)
M13F (-21)	TGTAAAACGACGGCCAGT	(Messing, 1983)
M13R	CAGGAAACAGCTATGAC	(Messing, 1983)

### *E. coli* cloning and sequencing

PCR products were ligated into the pCR4-TOPO vector and transformed into TOP10 *E. coli* cells following the manufacturer's instructions (Invitrogen, Carlsbad, CA). From each transformation event, eight resulting colonies were analyzed with the method of colony PCR. In brief, a single colony is transferred into 50 μL water, heated at 94°C for 5 min, then centrifuged for 30 s, and 1 μL of the supernatant is used as a template for PCR. After that, PCR was performed as previously described using the primers M13F (−21) (5′-TGTAAAACGACGGCCAGT-3′) and M13R (5′-CAGGAAACAGCTATGAC-3′) that bind to the vector sequence and amplify the cloned fragment. The PCR products were subjected to Sanger DNA sequencing in both directions at GeneWiz/Azenta, South Plainfield, NJ, USA.

### Sequence analysis and phylogenetic tree

Derived ITS sequences were analyzed using the software Geneious Prime version 2023.0.1 (Biomatters Ltd., Boston, MA, USA). The resulting contigs were analyzed for homology using the BLAST (Basic Alignment Search Tool) option and the NCBI nucleotide database. Resulting hits and ITS sequences for different species of *Tetraselmis* and *Chlorella* from the GenBank (Supplementary Tables 1, 2) were analyzed to characterize the variability between them. The sequences were aligned utilizing Geneious prime 2023.0.1 software to build the neighbor-joining phylogenetic tree using the Tamura-Nei genetic distance model with 500 replicates and no out-group to investigate the variation. A graphical representation of the sequence conservation was created using Geneious Prime, a sequence logo was created by WebLogo 3, and finally, a heatmap was created using GraphPad Prism. 8.

## Results

### Phylogenetic analysis and species identification using ITS markers

**Figure 2:**
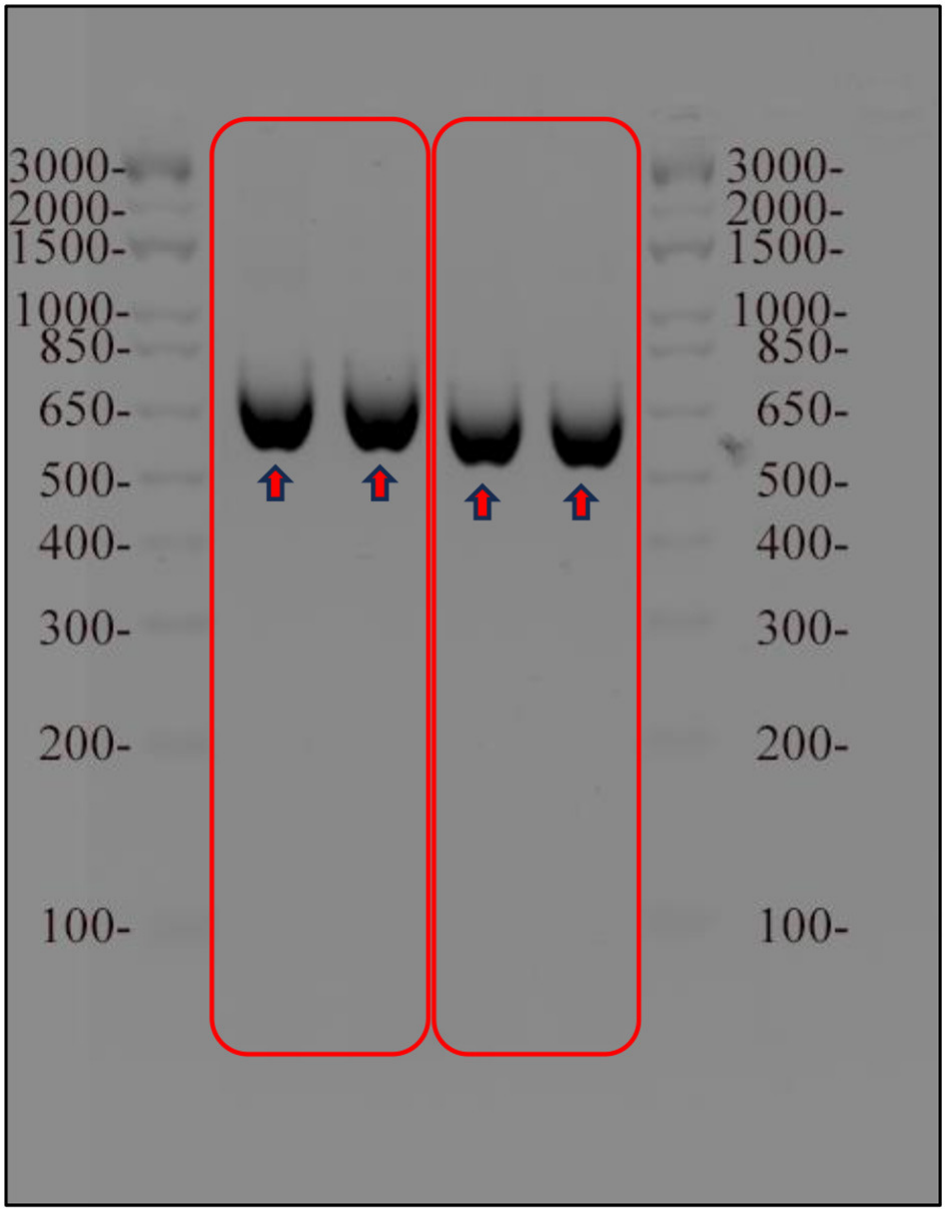
Results of agarose gel electrophoresis of the amplified ITS region of *Tetraselmis* and *Chlorella* samples. The ITS region was amplified using PCR and visualized on 1% agarose gel stained with ethidium bromide. A molecular weight marker was used as a reference for determining the size of the amplified ITS region for both samples.

In this study, the phylogeny and molecular diversity of the chlorophyta genera *Tetraselmis* and *Chlorella* were investigated. The main objective was to determine whether the internal transcribed spacer regions of the nuclear genome could serve as a reliable marker for species identification and differentiation of morphologically similar isolates, and which one is more accurate ITS1 or ITS2. To achieve this, DNA barcoding techniques were employed to compare the ITS markers of the isolates with the GenBank database. The resulting ITS PCR products were ~600 bps in length, as shown in Figure 2, gel electrophoresis. To confirm the sequences of the amplified regions, it was cloned into *E. coli* top 10 cells and conducted sequencing. Following sequencing, BLAST homology was performed on NCBI to determine the identities of the amplified ITS regions. The 612 base pairs long ITS PCR product derived from *Tetraselmis* transformants (NT3217) showed a 99.69% identity for the entire query length and was identified as *Tetraselmis* sp. KMMCC106 (accession number JQ315802). Similarly, the 593 base pairs long ITS PCR product derived from *Chlorella* transformants (NT3222) exhibited a 99.55% similarity and matched to *Chlorella* sp. SDEC-18 (accession number KY35143). The successful amplification of the full ITS genomic region was achieved using the ITS1 and ITS4 primers from extracted DNA. Our generated sequences have been deposited in the NCBI database, and they are assigned the respective NCBI accession numbers (OR120796 and OR120797).

**Figure 3:**
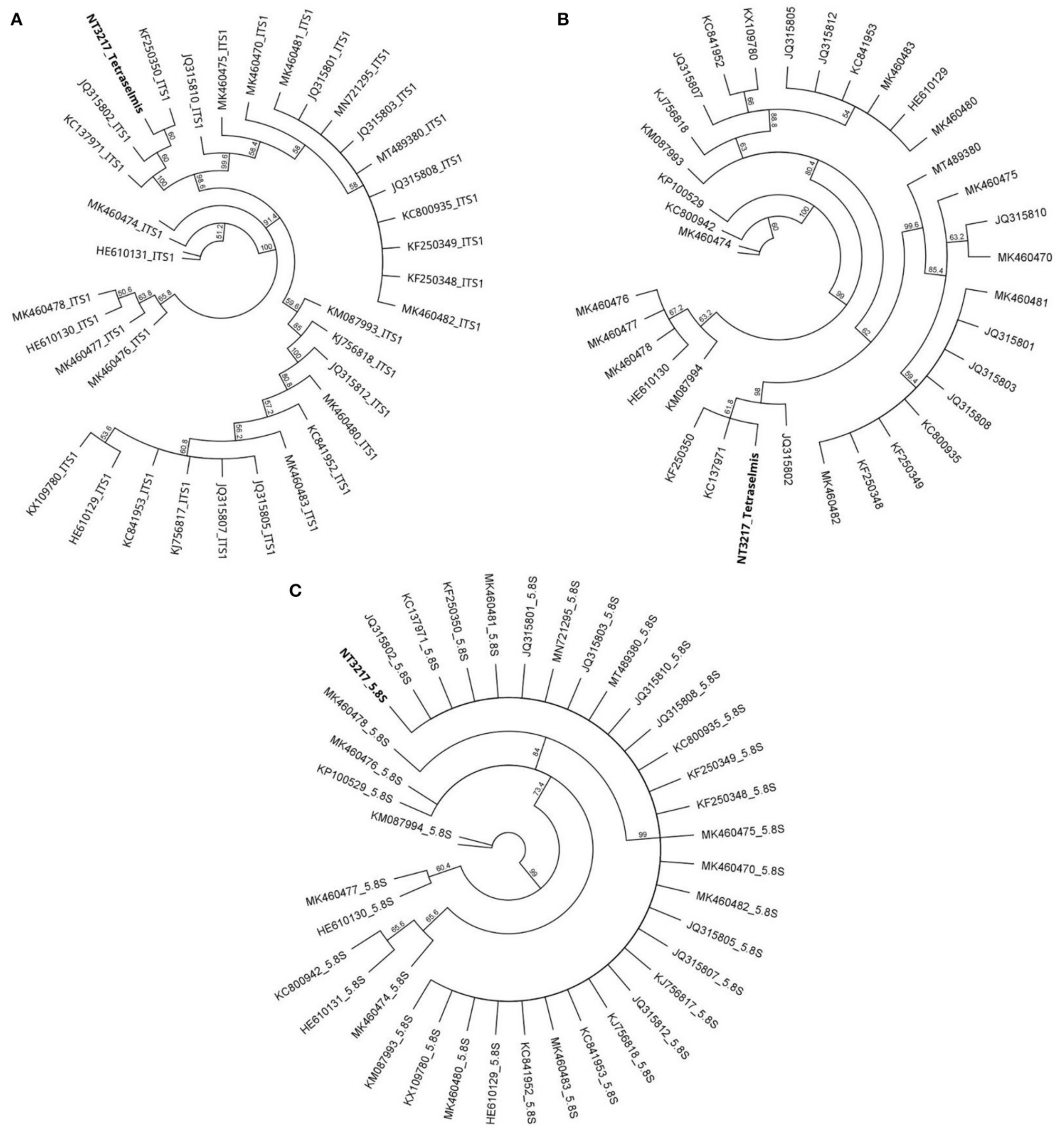
The evolutionary relationships among 38 distinct *Tetraselmis* sequences, as determined by the neighbor-joining method in a circular tree layout. Utilizing the Geneious Prime^®^ 2023.0.1 software, the figure presents separate phylogenetic trees for *Tetraselmis* ITS1 **(A)**, *Tetraselmis* ITS2 **(B)**, and *Tetraselmis* 5.8S **(C)**, offering a comprehensive analysis of the genetic diversity within the genus.

**Figure 4:**
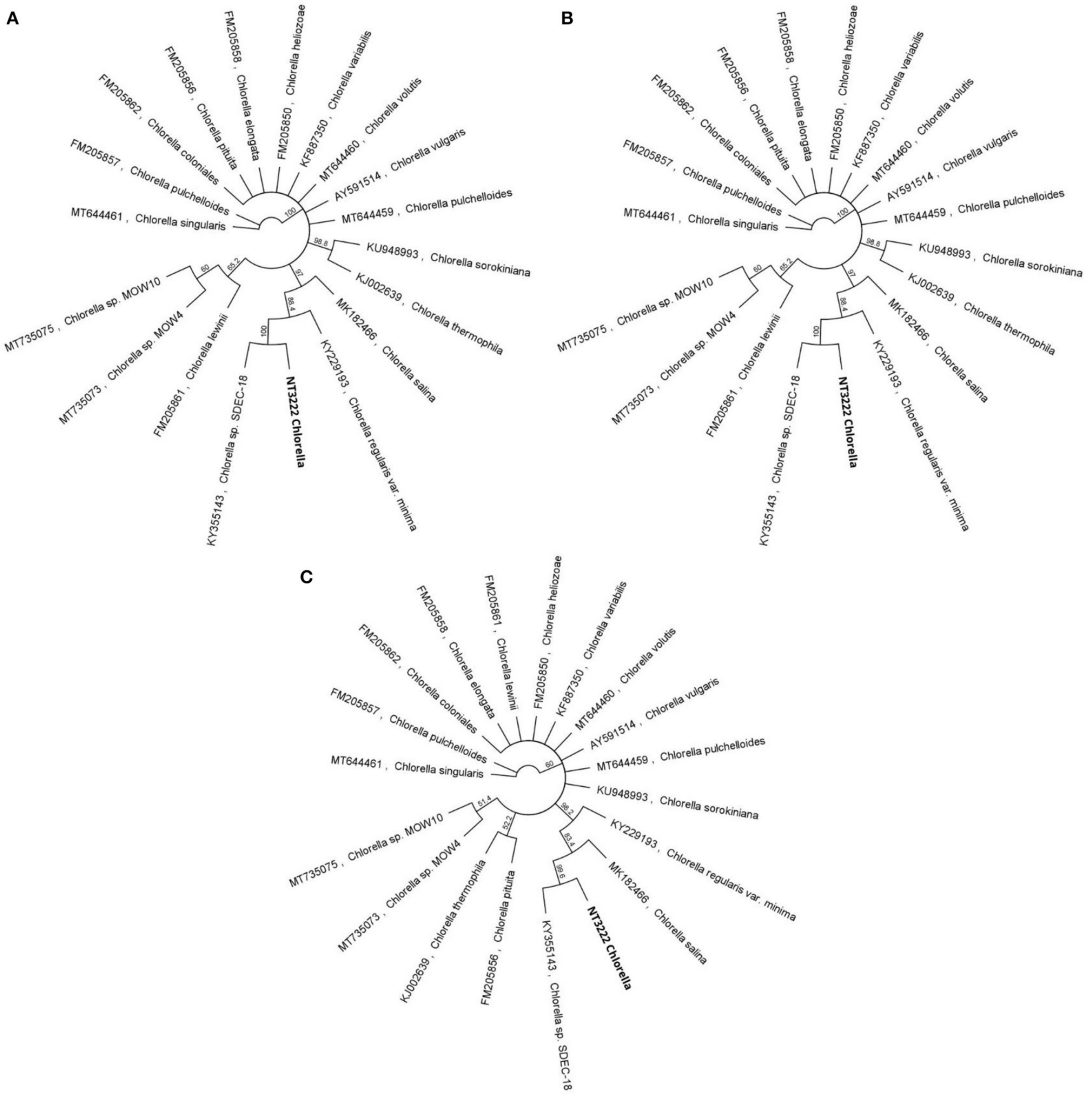
A compelling visual representation of the evolutionary relationships among 14 distinct *Chlorella* sequences, using the neighbor-joining method in a circular tree layout. By leveraging the advanced capabilities of the Geneious Prime^®^ software, the figure offers an in-depth examination of the genetic diversity within the genus, through separate phylogenetic trees for *Chlorella* ITS1 **(A)**, *Chlorella* ITS2 **(B)**, and *Chlorella* 5.8S **(C)** sequences.

Separate ITS-based phylogenetic trees incorporating 38 additional *Tetraselmis* and 14 additional *Chlorella* sequences were constructed for each taxon. The phylogenetic analysis of the *Tetraselmis* ITS1 and ITS2 sequences, depicted in Figure 3, yielded significant results. It was observed that the culture samples NT3217 and KC137971 as well as the sequence JQ315802 exhibited complete sequence identity. This suggests a tight relationship between these sequences, suggesting the existence of a possible clade or species group within *Tetraselmis*. Similarly, the phylogenetic analysis of the *Chlorella* ITS1 and ITS2 sequences, described in Figure 4, yielded insightful information. We discovered that isolate sequence NT3222 and *Chlorella* sp. SDEC-18 (accession KY35143) shared 100% sequence identity. This suggests that these sequences may share a common ancestry or species affiliation within *Chlorella* due to their high degree of similarity. These results demonstrate the significance of the ITS1 and ITS2 regions for phylogenetic analyzes of *Tetraselmis* and *Chlorella* species. The observed sequence identities between the examined samples and other sequences provide valuable evidence for comprehending the genetic relationships and possible classifications within these genera.

### Alignment analysis of ITS1, ITS2, and 5.8S regions

**Figure 5:**
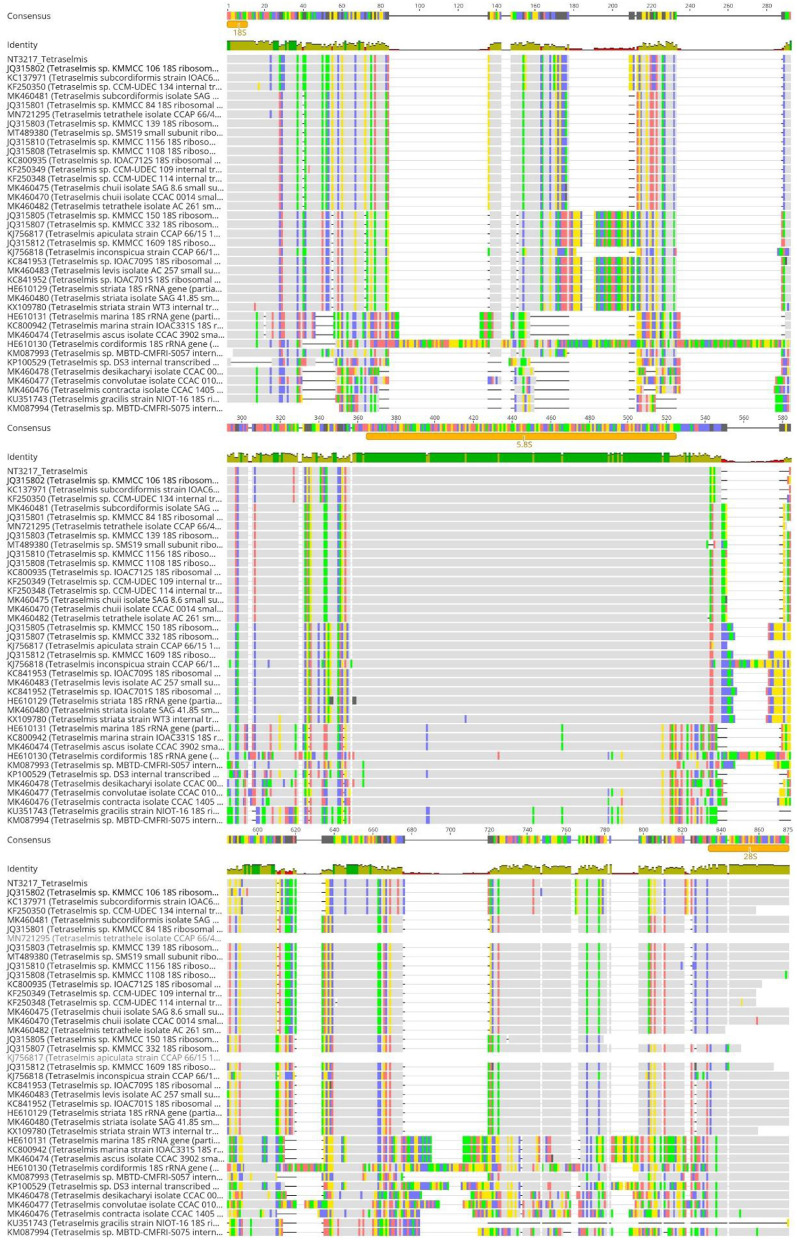
Alignment of ITS sequences from various *Tetraselmis* species using Geneious Prime. The consensus threshold was set to 65% similarity. Sequence NT3217 was derived from the analyzed *Tetraselmis* isolate. The highest sequence identity of the analyzed isolate was *Tetraselmis subcordiformis* and *Tetraselmis* sp. KMMCC106 (accessions KC137971 and JQ315802). Consensus identity colors: green stands for 100% identity, sites with 30% to under 100% identity are yellow, and sites with <30% identity are marked in red. White or non-colored areas are identical.

The alignments of the ITS1, ITS2, and 5.8S regions are crucial for investigating the differentiation between these sequences, in addition to assessing the identity and similarity within the *Tetraselmis* and *Chlorella* genera. Regarding the *Tetraselmis* genus, the ITS1 alignment revealed a high degree of sequence identity among the culture sequences NT3217 to KC137971 and JQ315802, with a value of 100%, as shown in Figure 5. Additionally, a relatively high degree of sequence similarity, with a value of 99.51%, was observed between culture sequence NT3217 and KF250350. The sequence identity among other species within the genus ranged from 31.25% to 87.32% in the ITS1 alignment. While similar patterns were observed in the ITS2 region, where the culture sequences NT3217 and KC137971 exhibited 100% sequence identity, the sequence identity between NT3217 and KF250350 was 99.49%. The sequence identity among other species within the ITS2 alignment ranged from 34.01% to 80.20%. However, alignment of the 5.8S region showed that culture sequence NT3217 shared 100% sequence identity with many other *Tetraselmis* species, while the sequence identity between different species ranged from 91.93% to 100%. These results indicate that the ITS1 and ITS2 regions are useful for distinguishing between cultures and other species within the *Tetraselmis* genus, while the 5.8S region did not provide significant distinctions.

**Figure 6:**
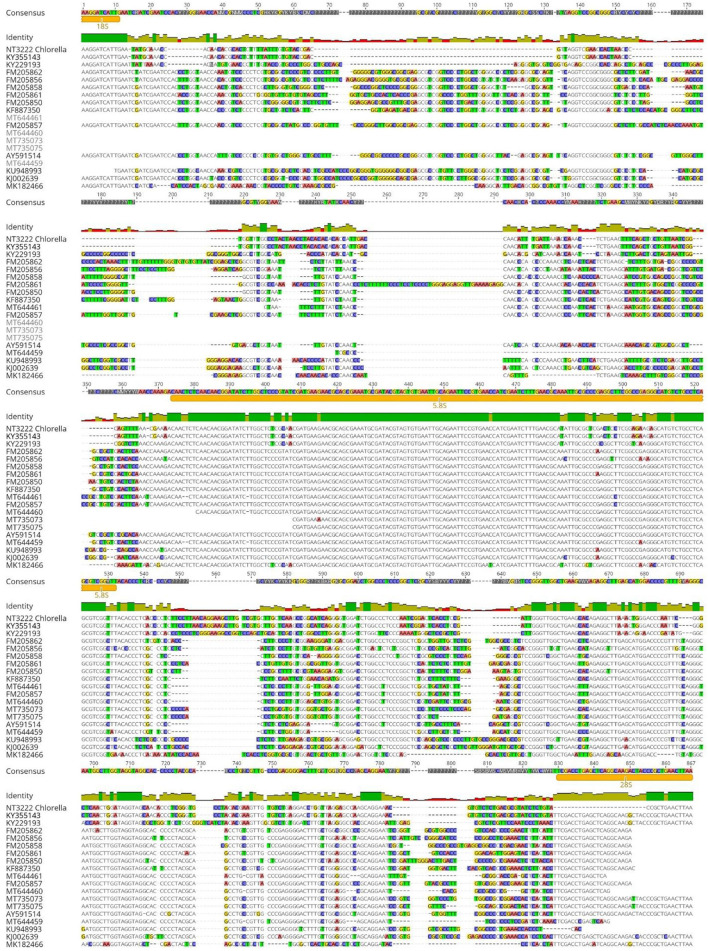
Alignment of ITS sequences from various *Chlorella* species using Geneious Prime. The consensus threshold was set to 65% similarity. Sequence NT3222 was derived from the analyzed *Chlorella* isolate. The highest sequence identity of the analyzed isolate was *Chlorella* sp. SDEC-18 with a 100% sequence identity (accession KY355143.1). Consensus identity colors: green stands for 100% identity, sites with 30% to under 100% identity are yellow, and sites with <30% identity are marked in red. White or non-colored areas are identical.

For the *Chlorella* genus, the ITS1 alignment demonstrated a high degree of sequence identity between culture sequences NT3222 and KY35143, with a value of 100% as shown in Figure 6. However, the ITS1 alignment also revealed a relatively low degree of sequence identity between different species within the genus, ranging from 11.39% to 43.58%. Similarly, the ITS2 region exhibited 100% sequence identity between culture sequences NT3222 and KY35143, while the sequence identity between different species ranged from 37.16% to 59.62%. In contrast, the alignment of the 5.8S region yielded different results, with little reliable species identification and a low degree of nucleotide change observed between different species. Overall, these findings suggest that the ITS1 and ITS2 regions are useful for distinguishing between culture sequences NT3222 and KY35143 and different species within the *Chlorella* genus, while the 5.8S region does not provide as much information for this purpose.

### Sequence variation analysis of ITS regions utilizing sequence logos

**Figure 7:**
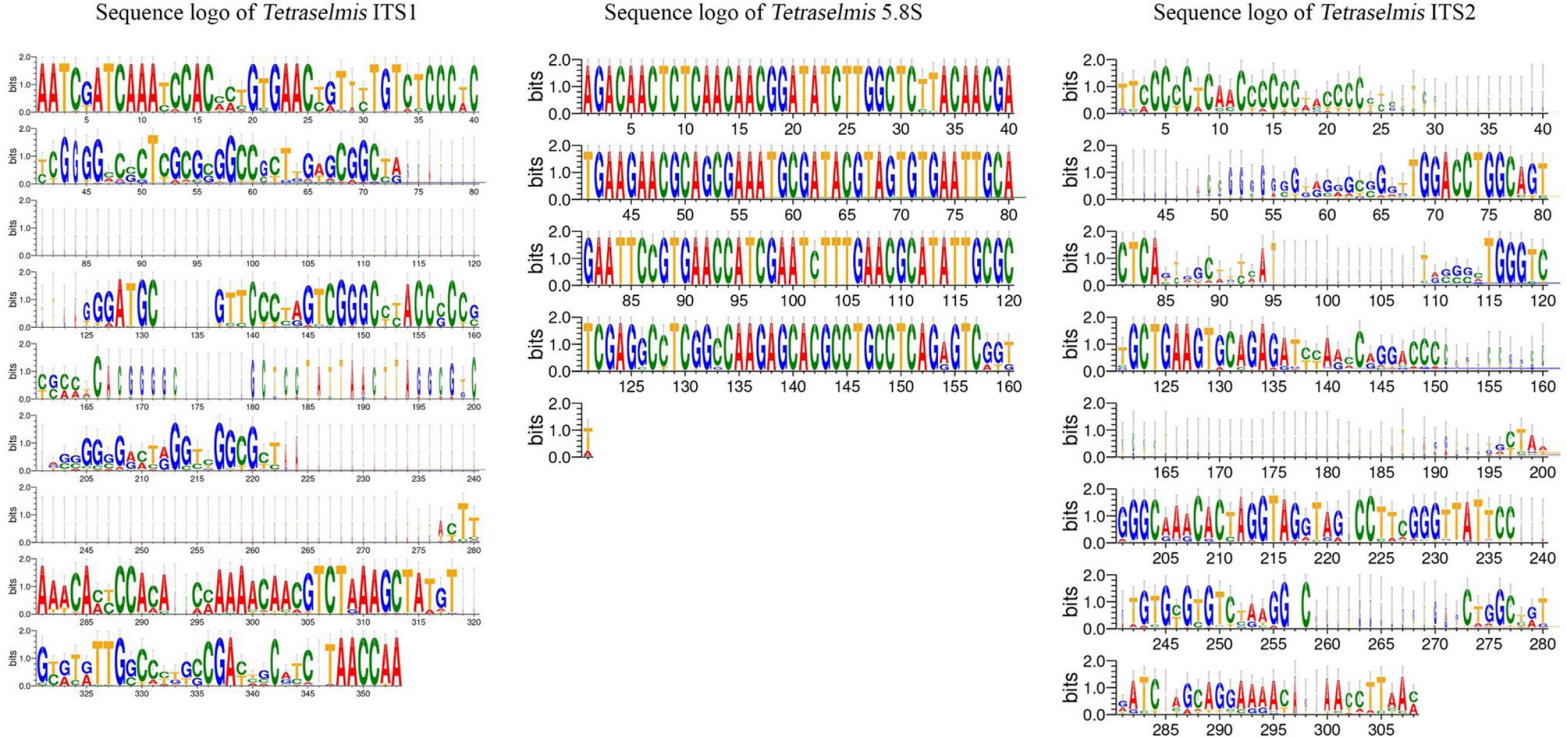
Sequence logo created from the *Tetraselmis* ITS1, 5.8S, and ITS2 multiple DNA sequence alignment. Every logo is made up of a collection of aligned sequences. The height of each letter within the logo represents the relative frequency of a nucleotide at that place, while the height of the letter represents the conservation of the sequence at that position (Crooks et al., 2004).

**Figure 8:**
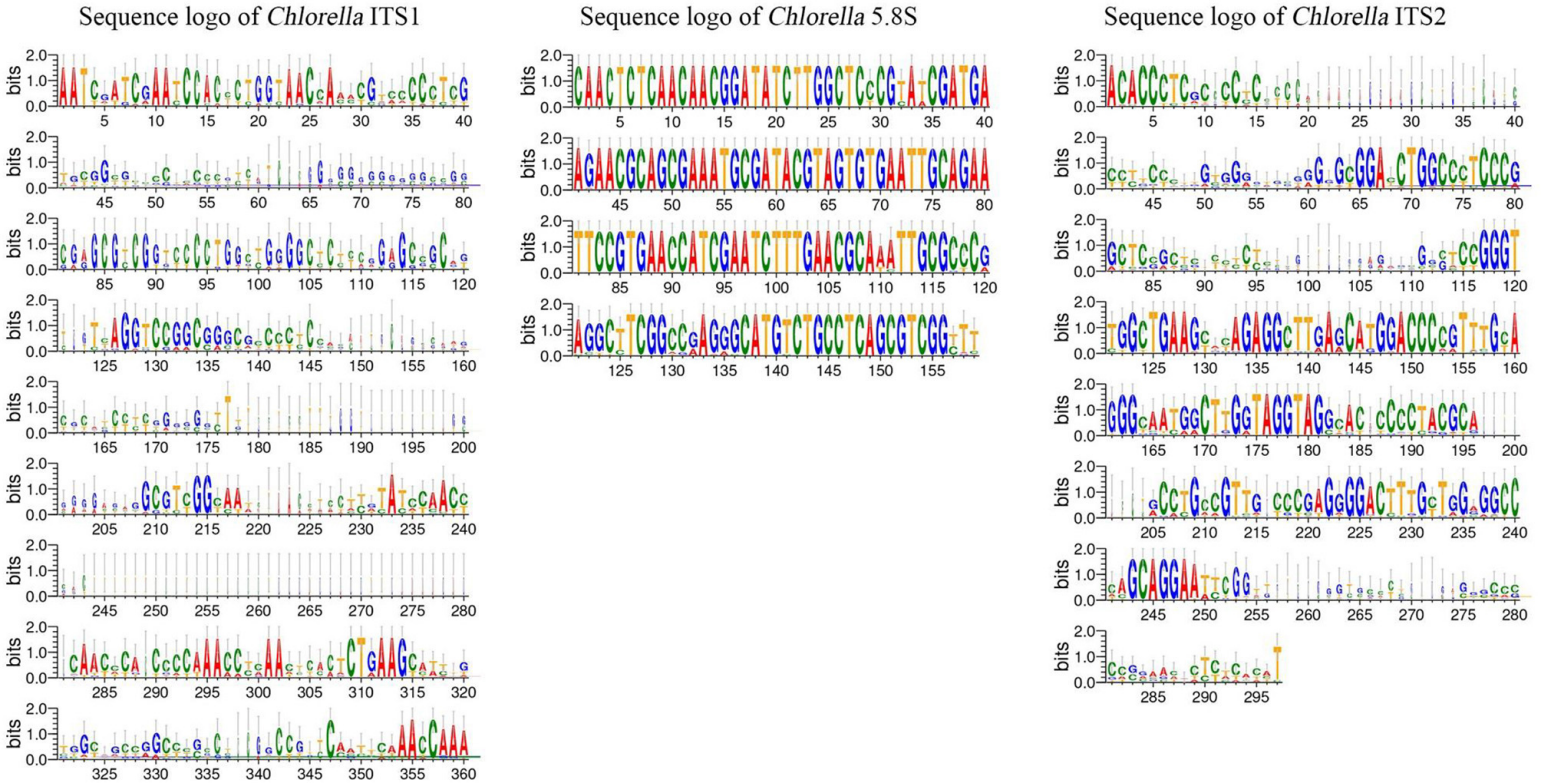
Sequence logo created from the *Chlorella* ITS1, 5.8S, and ITS2 multiple DNA sequence alignment. Every logo is made up of a collection of aligned sequences. The height of each letter within the logo represents the relative frequency of a nucleotide at that place, while the height of the letter represents the conservation of the sequence at that position (Crooks et al., 2004).

The ITS1 and ITS2 regions of the *Chlorella* and *Tetraselmis* genera were aligned to determine the degree of sequence variation. The sequence logos generated for *Tetraselmis* (Figure 7) and *Chlorella* (Figure 8) reveal that the ITS2 region displayed a greater degree of sequence variation than the ITS1 region, as revealed by the analysis. Sequence logos are graphical representations obtained from DNA multiple-sequence alignments. They provide valuable insight into the evolutionary and functional relationships between sequences. The sequence logos displayed here illustrate the conservation and relative frequency of nucleotides at each position within aligned regions. The height of the logo's letters corresponds to the degree of conservation and frequency, facilitating the interpretation of functional and evolutionary relationships between sequences.

### Assessment of sequence homology and interspecific variation using heatmaps

**Figure 9:**
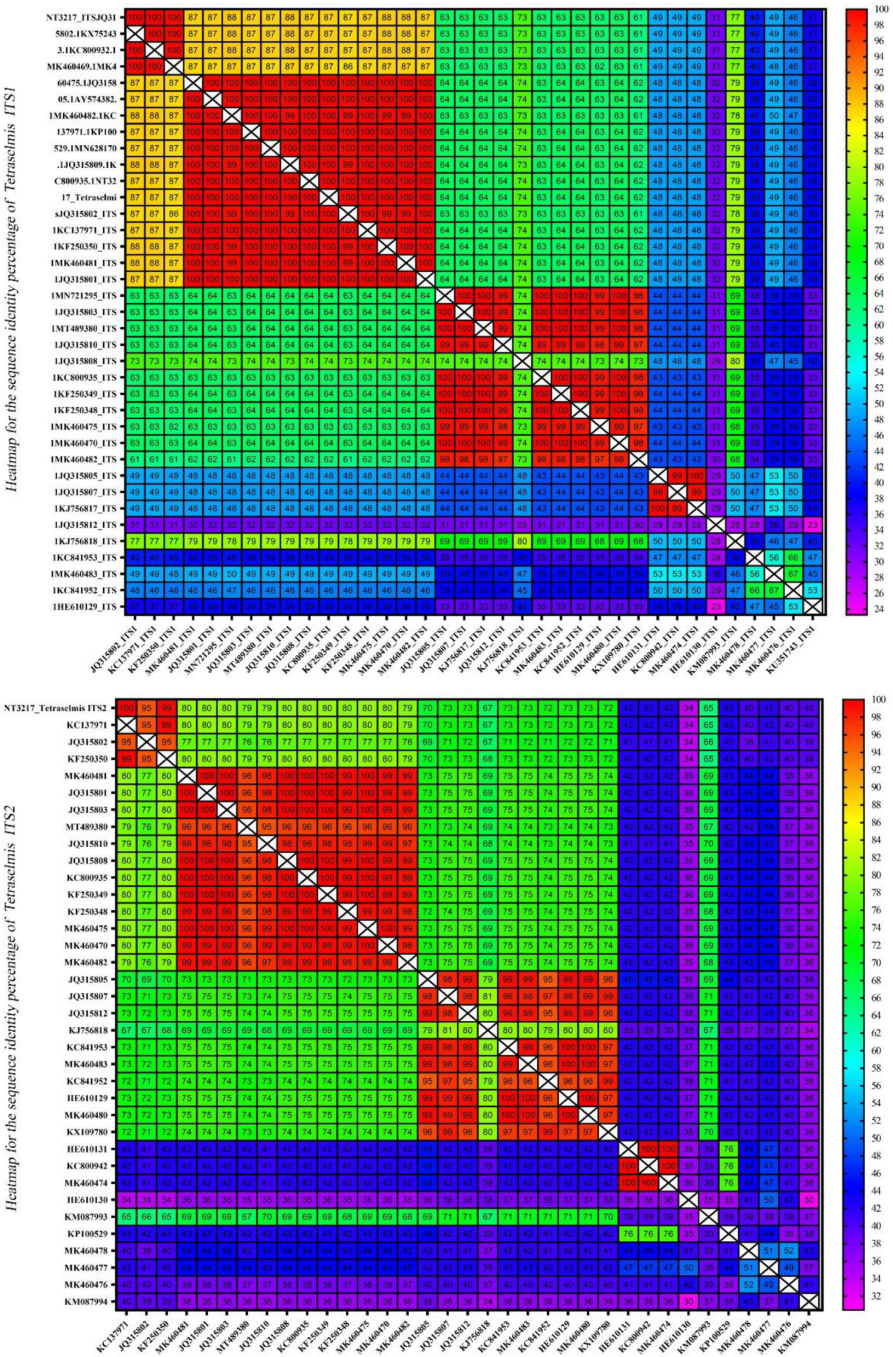
A heatmap representing the sequence identity percentage of the ITS region of 38 *Tetraselmis* sequences, including an isolate (NT3217) which was investigated in this study. The heatmap uses color coding to indicate the degree of similarity between the ITS sequences, with red indicating a high degree of identity. This heatmap provides a visual representation of the sequence identity percentage, enabling easy comparison and identification of the ITS sequences.

**Figure 10:**
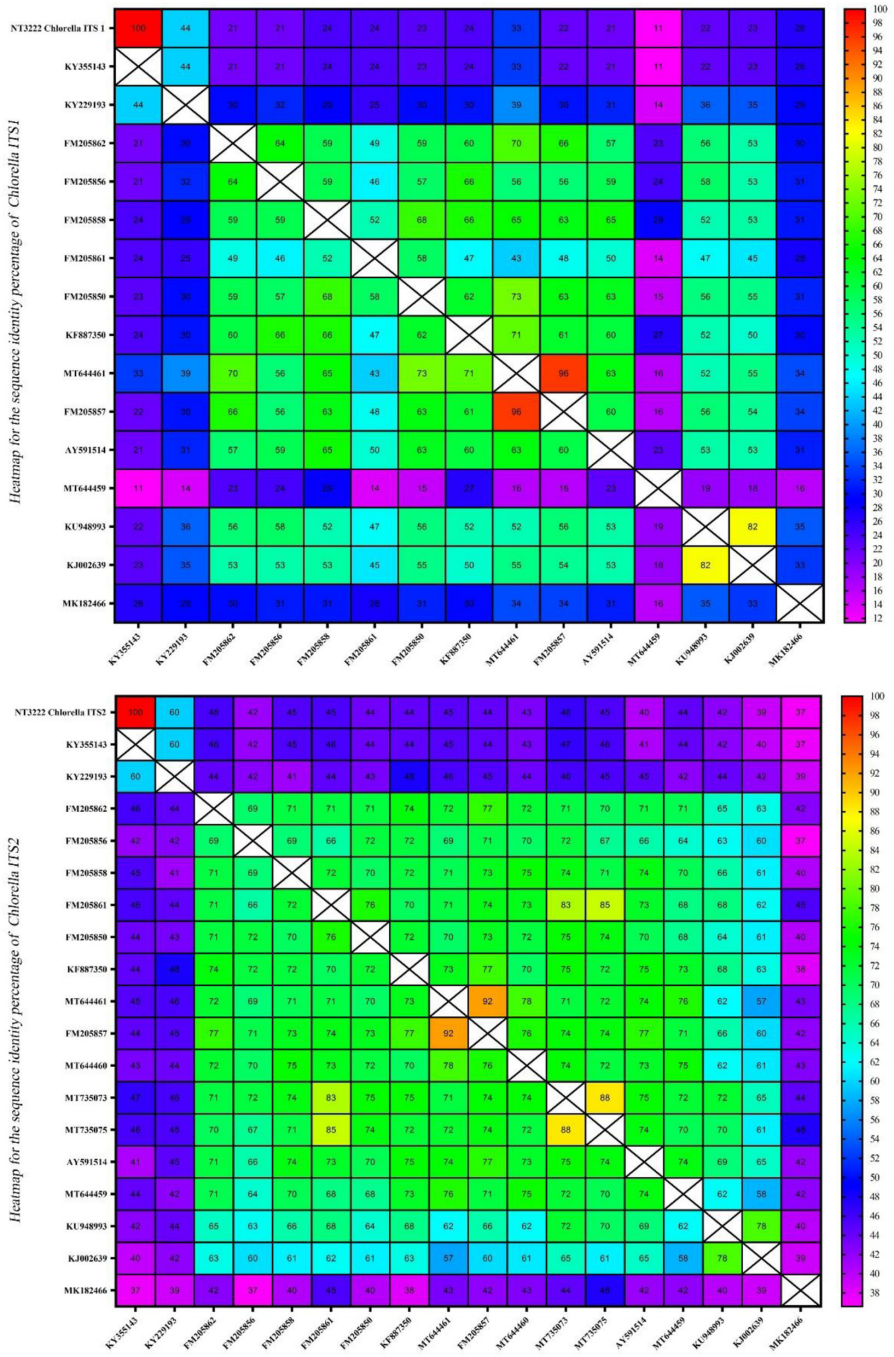
A heatmap representation of the sequence identity percentage of the ITS region of 14 different *Chlorella* sequences and one *Chlorella* isolate (NT3222) that was specifically investigated in this study. This heatmap provides a visual representation of the sequence identity percentage, allowing for easy comparison and identification of the ITS sequences.

The homology of the ITS1 and ITS2 regions in the microalgae *Tetraselmis* and *Chlorella* was examined. To evaluate sequence similarity, heatmaps were used to depict the proportion of identical base pairs in each ITS1 and ITS2 sequence. These heatmaps shed light on the degree of similarity and potential misidentifications among the species. The heatmaps generated for *Tetraselmis* (Figure 9) revealed a striking pattern of high identity, with values exceeding 97%, among the numerous *Tetraselmis* species. This finding raises questions about the accuracy of species identification, indicating the possibility of misidentification in certain instances. To ensure accurate taxonomic assignments within the *Tetraselmis* genus, additional research and validation are necessary. While Figure 10 depicts the distinct pattern exhibited by the *Chlorella* heatmap, the preponderance of observed identities was < 90% indicating a high degree of interspecific variation among *Chlorella* species. This result suggests that *Chlorella* species exhibit a greater degree of genetic diversity and differentiation. The observed interspecific variation demonstrates the importance of meticulous consideration and exhaustive analysis when classifying and identifying *Chlorella* species. Overall, these results reveal a high degree of identity among *Tetraselmis* species and substantial interspecific variation among *Chlorella* species, as indicated by the heatmaps generated for the ITS1 and ITS2 regions. These findings emphasize the importance of precise species identification and further exploration of genetic diversity within these algal genera.

## Discussion

### Applying DNA barcoding in taxonomy to investigate the diversity of microalgae

It is currently assumed that there exist over 72,500 species of algae across the globe, with only 60.7% of them having been formally documented (Guiry, 2012). With numerous undiscovered species still awaiting identification, the development of a new and efficient system for describing algae becomes imperative. With ~8,000 described species, the green algae, scientifically known as chlorophyta, form an ancient and taxonomically diverse lineage. However, it is believed that a substantial number of species, ~62.5%, that are primarily concentrated in tropical and subtropical areas remain undescribed (Guiry, 2012). Therefore, DNA barcoding plays a crucial role in overcoming the limitations of traditional species identification methods. However, this does not diminish the importance of traditional taxonomy. On the contrary, DNA barcoding has emerged as a valuable tool for taxonomists, complementing their existing knowledge and providing an innovative means for non-experts to rapidly identify organisms. Akter et al. (2023) reported that DNA barcoding is a valuable and advanced scientific method used for various applications in species identification and biodiversity research. It serves as an ideal tool for species discovery and identification, facilitating the recognition of difficult-to-identify species. DNA barcoding can be applied to fish, algae, plants, and animal species. Additionally, it plays a significant role in identifying species with ambiguous morphological features, ensuring a safe food chain and industry. Moreover, DNA barcoding helps ensure food authenticity, preventing food fraud and maintaining the integrity of the food chain and economic status of a country. However, when it comes to microalgae and protists, which display a higher level of genetic diversity compared to the aforementioned groups, there is an ongoing discussion regarding the identification of suitable markers. In most phytogeographic studies, ITS has been extensively employed. Located between the small and large ribosome subunits, the ITS region is a non-coding domain. It has gained popularity due to its relatively high nucleotide substitution rate, enabling comparisons among taxa that have recently diverged. The ITS region is easily amplified through PCR and can be sequenced using conservative primers. It has been proposed as a potential barcode for both algae and terrestrial plants, finding widespread use in the phylogenetics of green algae species. The ITS1 and ITS2 markers exhibit sufficient variability to distinguish between different strains of algae (Kowalska et al., 2019).

### Multiple analysis approaches for accurate species identification

Through investigation, Table 2 presents the results of different analysis methods used to identify species codes for *Tetraselmis* and *Chlorella*, along with the corresponding matched strains and identification percentages, via comparing the various analysis methods and discussing their effectiveness. NCBI Blast analysis yielded high identification percentages for both *Tetraselmis* (99.69%) and *Chlorella* (99.55%) using the ITS marker. This method compares the query sequences with sequences in the NCBI database, providing reliable matches. It is a commonly used and effective tool for species identification. The phylogenetic analysis resulted in perfect matches (100%) for both *Tetraselmis* (NT3217) and *Chlorella* (NT3222) using the ITS marker. This method reconstructs evolutionary relationships based on genetic data, and in this case, it successfully determined the relatedness between the analyzed strains. Sequence alignment analysis showed high identification percentages for *Tetraselmis* and *Chlorella* using different markers. ITS1 and ITS2 markers provided good matches for *Tetraselmis* (NT3217) and *Chlorella* (NT3222), with some variations in percentages. The matches with additional strains also revealed the presence of closely related species. The 5.8S marker exhibited a match (100%) for many *Tetraselmis* sp. and many *Chlorella* sp., indicating their similarity in this region. Heatmap analysis indicated a 97% identification for *Tetraselmis* (NT3217) and a lower identification percentage (< 90%) for *Chlorella* (NT3222) when compared to more than three strains. The heatmap analysis provides a visual representation of the similarity between samples based on genetic data. In conclusion, each analysis method has its strengths and limitations. It is recommended to utilize multiple analysis methods to enhance the accuracy and reliability of species identification. Combining NCBI Blast analysis, which compares sequences to a comprehensive database, with phylogenetic analysis and sequence alignment using different markers allows for a more comprehensive understanding of the genetic relationships and species identification. The heatmap analysis can provide additional insights into the similarity between samples. By employing a combination of these analysis methods, researchers can strengthen the validity of their results and gain a deeper understanding of the genetic diversity and relatedness of species within the studied groups. This conclusion, which is supported by González et al. (2013), showcases the importance of using a multidisciplinary approach, combining ultrastructural analysis, genetic sequencing, and phylogenetic tools, to address taxonomic challenges and evaluate the biotechnological potential of microalgae strains.

**Table 2:** Comparative analysis results for species identification in *Tetraselmis* and *Chlorella* using different methods and markers.

**Analysis type**	**Species**	**Code**	**Matched strain**	**Identification percentage**	**Target**
NCBI Blast	*Tetraselmis*	NT3217	JQ315802	99.69%	ITS
	*Chlorella*	NT3222	KY35143	99.55%	ITS
Phylogenetic analysis	*Tetraselmis*	NT3217	KC137971	100%	ITS
			JQ315802		
	*Chlorella*	NT3222	KY35143	100%	ITS
Sequence alignment	*Tetraselmis*	NT3217	KC137971	100%	ITS1
			JQ315802		
			KF250350	99.51%	ITS1
			KC137971	100%	ITS2
			KF250350	99.49%	ITS2
			Many *Tetraselmis* sp.	100%	5.8S
	*Chlorella*	NT3222	KY35143	100%	ITS1
			KY35143	100%	ITS2
			Many *Chlorella* sp.	100%	5.8S
Heatmap	*Tetraselmis*	NT3217	More than 3 strains	97%	ITS
	*Chlorella*	NT3222	More than 3 strains	< 90%	ITS

In a nutshell, this study covered the full ITS region, which comprises three key segments (ITS1, 5.8S, and ITS2) that are particularly useful for interspecific variation analysis. The findings suggest that the ITS region can serve as a powerful genomic marker for broad application in the field of microalgae research (Moreira et al., 2016). These findings confirm that both ITS1 and ITS2 can be used in identifying different species of chlorophyta based on a sequence length ranging between ~200 bp and ~300 bp. This assumption is supported by previous research conducted by Hall et al. (2010), in which numerous DNA barcoding markers for green algae (SSU, UPA, rbcL, tufA, and coxI) were evaluated. The study found that rbcL, ITS2, and tufA were the most promising markers for use as barcodes in green algae. Additionally, Chu et al. (2001) demonstrated that ITS1 variations are prominent among different taxonomic groups of crustaceans, and variations between congeneric species appear to be genus-specific. This makes ITS1 a valuable molecular marker for phylogenetic analysis in some genera and a useful diagnostic tool in others. The use of ITS regions as barcodes for species identification in chlorophyta aligns with the increasing trend of molecular identification in modern taxonomy, which allows for faster and more accurate identification of species, especially for those that are difficult to distinguish morphologically. The advantages of ITS regions as barcode markers, in particular ITS2, are their universality, high level of variation, and easy amplification by PCR. Furthermore, these results indicate that ITS2 is more informative than ITS1 and 5.8S. This can help to identify new unknown species, resolve conflicting identifications, and expand the use of DNA barcoding to the other taxa in green algae. Based on these results, it can be concluded that the ITS region can be considered an effective molecular marker for describing species variation within the microalgae (Yoshida et al., 2008).

### Integrating image comparison and DNA barcoding as a future perspective for establishing a digital library for microalgae identification

The chlorophyta phylum, comprising a diverse array of green microalgae, has garnered significant attention in recent years due to its vast array of biotechnological applications. These microorganisms have been identified as having enormous potential for use in the pharmaceutical, food, and biofuel industries, primarily due to their ability to produce valuable compounds such as fatty acids, polyunsaturated fatty acids, and lipids (Fathy et al., 2022). Furthermore, chlorophyta have been found to play a significant role in environmental industries, with notable examples including the biodegradation of pesticide contaminants and the removal of heavy metals from contaminated environments. Given the importance of these microorganisms, the identification and characterization of different chlorophyta strains are of paramount importance. However, the small size and morphological plasticity of microalgae can make this task challenging, and traditional identification methods can often prove inadequate. DNA barcoding, a powerful molecular technique, offers a reliable and accurate method for identifying microalgae species (Mustafa et al., 2021). This method can be particularly useful in differentiating closely related species. Overall, the combination of DNA barcoding with other analytical techniques offers a potent tool for uncovering microalgae biodiversity and plays a critical role in advancing our understanding of these important microorganisms (González et al., 2015).

Consequently, in the field of microalgae identification, there is an increasing demand for efficient and precise species recognition techniques. The establishment of a digital library for the identification of microalgae based on image comparison is a potential solution to this problem. Allowing for the integration of morphological and genetic data, this novel method could substantially improve the speed and accuracy of species identification. The proposed digital library would serve as a comprehensive repository for images of microalgae, encompassing a broad variety of species and their morphological characteristics. Users would be able to upload images of unidentified microalgae specimens, which would then be compared to images in the library using sophisticated image recognition algorithms. Utilizing machine learning and pattern recognition techniques, the system can rapidly analyze uploaded images and propose potential matches based on visual similarities. After obtaining potential species matches via image comparison, the system could validate the identification by conducting DNA barcoding. DNA barcoding will be done by comparing the genetic sequence of a particular region, such as the ITS, and comparing it with known sequences in a DNA database in NCBI. By incorporating DNA barcoding into the identification process, the accuracy of species identification would be significantly improved, resulting in a comprehensive and trustworthy identification result. Numerous benefits can be associated with this strategy. It will provide a user-friendly and accessible platform for microalgae identification, allowing researchers, students, and even non-specialists to identify microalgae strains rapidly and accurately. It will considerably reduce the dependence on taxonomic expertise, which is frequently limited and time-consuming. In addition, by combining morphological and genetic data, any misidentified or mislabeled specimens will be identified, resulting in enhanced data quality and reliability. The establishment of a digital library for microalgae taxonomy and identification based on image comparison and DNA barcoding holds tremendous promise for advancing the field of microalgae taxonomy and identification. By integrating morphological and genetic data in a user-friendly and accessible platform, this method can revolutionize the way microalgae are identified, enhancing data accuracy, and facilitating further research in diverse fields such as ecology, biotechnology, and environmental monitoring. Our concept agreed with the study of Chong et al. (2023), which is focused on the integration of image processing and machine learning to improve microalgae species identification. Specifically, advanced image processing techniques, including deep learning algorithms, are advocated to enhance the accuracy and efficiency of the identification process. Additionally, the study emphasizes the incorporation of various image pre-processing modules to enhance image quality by removing unwanted artifacts and background noise. Furthermore, the article highlights the importance of machine learning algorithms such as artificial neural networks, support vector machines, and convolutional neural networks in achieving reliable image classification for microalgae identification. The review also discusses future possibilities, where a robust digital classification tool using machine learning and image processing can be developed to address challenges associated with conventional identification methods.

## Conclusion

In this study, we aimed to assess the potential of DNA barcoding as a standardized method to identify chlorophyta microalgae, which are crucial for producing diverse bioproducts, including biofuels, high-value bioactive proteins, and dietary supplements. The traditional approach of morphological identification under a microscope is challenging, time-consuming, and prone to inaccuracies. To achieve our objective, we analyzed the ITS genomic region of *Chlorella* and *Tetraselmis* isolates and compared them with reference sequences from 38 *Tetraselmis* and 14 *Chlorella* species. Our investigation demonstrated that the ITS region serves as a valuable tool for distinguishing and identifying morphologically similar chlorophyta species. Notably, ~200–300 base pairs of the ITS1 and ITS2 sections were found sufficient for differentiating the isolates from each other. Particularly, the ITS2 DNA region exhibited a higher identification efficiency than the ITS1 region due to its greater interspecific variation. These findings indicate that ITS2 can serve as a useful DNA barcode for identifying a wide range of chlorophyta species. While DNA barcoding proves to be a significant advancement for species identification, it is essential to enhance accuracy and reliability by integrating additional genetic markers or molecular techniques. Incorporating other markers, such as chloroplast-targeting primers or sequence analysis of other genomic regions, can establish a more robust and comprehensive species identification system. Furthermore, considering the practical applicability of using the ITS region as a standalone marker for species differentiation is crucial. Future research should integrate image processing and machine learning to improve microalgae species identification. A comparative analysis will provide a better assessment of the reliability and accuracy of employing the ITS region alone for species differentiation. Despite the challenges, DNA barcoding remains a crucial tool for combating species misidentification. Advancements in sequencing technology, curated databases with species descriptions, and taxonomic clarifications offer promising prospects for improving eukaryotic microalgae identification in the future. Moreover, applying next-generation sequencing approaches can further expand the potential of DNA barcoding in library preparation and accurate identification in biodiversity research.

## Data availability statement

The datasets presented in this study can be found in online repositories. The names of the repository/repositories and accession number(s) can be found in the article/Supplementary material.

## Author contributions

WF was involved in conceptualization, data analysis, and manuscript writing. NT contributed to data analysis, interpretation, and manuscript revision. KE participated in data collection, analysis, and manuscript editing. EE provided support in data analysis and interpretation, as well as manuscript review. ET assisted in data collection, analysis, and manuscript editing. KA contributed to data interpretation and manuscript revision. MA was involved in study design, data analysis, and manuscript writing. OH and SR provided guidance in data analysis, interpretation, and manuscript review. All authors contributed to the article and approved the submitted version.
