# Supplementary material for: Applying an internal transcribed spacer as a single molecular marker to differentiate between Tetraselmis and Chlorella species
Source: Front Microbiol. 2023 Aug 23;14:1228869. doi: 10.3389/fmicb.2023.1228869 (PMC10482269; doi:10.3389/fmicb.2023.1228869)

Appling an internal transcribed spacer as a single molecular marker to differentiate between *Tetraselmis* and *Chlorella* species.

Wael A. Fathy[1, *], Natascha Techen[2], Khaled N.M. Elsayed[1], Ehab Essawy[3], Eman Tawfik[4], Khairiah Mubarak Alwutayd[5]. Mohamed S. Abdelhameed[1], Ola Hammouda[1], Samir A. Ross[2,6]

Supporting data:

Table 1. *Tetraselmis* ITS sequences used for the alignment. (All the tabulated sequences are adopted from the NCBI https://www.ncbi.nlm.nih.gov/nuccore)

NO.	Sequence name/ accession number	Species
1	JQ315802.1	*Tetraselmis* sp. KMMCC 106
2	KC137971.1	*Tetraselmis subcordiformis* strain IOAC677S
3	KF250350.1	*Tetraselmis* sp. CCM-UDEC 134
4	HE610129.1	*Tetraselmis striata*
5	HE610130.1	*Tetraselmis cordiformis*
6	HE610131.1	*Tetraselmis marina*
7	JQ315801.1	*Tetraselmis* sp. KMMCC 84
8	JQ315803.1	*Tetraselmis* sp. KMMCC 139
9	JQ315805.1	*Tetraselmis* sp. KMMCC 150
10	JQ315807.1	*Tetraselmis* sp. KMMCC 332
11	JQ315808.1	*Tetraselmis* sp. KMMCC 1108
12	JQ315810.1	*Tetraselmis* sp. KMMCC 1156
13	JQ315812.1	*Tetraselmis* sp. KMMCC 1609
14	KC800935.1	*Tetraselmis* sp. IOAC712S
15	KC800942.1	*Tetraselmis marina* strain IOAC331S
16	KC841952.1	*Tetraselmis* sp. IOAC701S
17	KC841953.1	*Tetraselmis* sp. IOAC709S
18	KF250348.1	*Tetraselmis* sp. CCM-UDEC 114
19	KF250349.1	*Tetraselmis* sp. CCM-UDEC 109
20	KJ756817.1	*Tetraselmis apiculata* strain CCAP 66/15
21	KJ756818.1	*Tetraselmis inconspicua* strain CCAP 66/19C
22	KM087993.1	*Tetraselmis* sp. MBTD-CMFRI-S057
23	KM087994.1	*Tetraselmis* sp. MBTD-CMFRI-S075
24	KP100529.1	*Tetraselmis* sp. DS3
25	KU351743.1	*Tetraselmis gracilis* strain NIOT-16
26	KX109780.1	*Tetraselmis striata* strain WT3
27	MK460470.1	*Tetraselmis chuii* isolate CCAC 0014
28	MK460474.1	*Tetraselmis ascus* isolate CCAC 3902
29	MK460475.1	*Tetraselmis chuii* isolate SAG 8.6
30	MK460476.1	*Tetraselmis contracta* isolate CCAC 1405
31	MK460477.1	*Tetraselmis convolutae* isolate CCAC 0100
32	MK460478.1	*Tetraselmis desikacharyi* isolate CCAC 0029
33	MK460480.1	*Tetraselmis striata* isolate SAG 41.85

34	MK460481.1	*Tetraselmis subcordiformis* isolate SAG 161.1a
35	MK460482.1	*Tetraselmis tetrathele* isolate AC 261
36	MK460483.1	*Tetraselmis levis* isolate AC 257
37	MN721295.1	*Tetraselmis tetrathele* isolate CCAP 66/41
38	MT489380.1	*Tetraselmis* sp. SMS19

Table. 2 *Chlorella* ITS sequences used for the alignment. (All the tabulated sequences are adopted from the NCBI https://www.ncbi.nlm.nih.gov/nuccore)

NO.	Sequence name/ accession number	Species
1	KY355143.1	*Chlorella* sp. SDEC-18
2	FM205858.1	*Chlorella* sp. CCAP 222/18
3	FM205861.1	*Chlorella lewinii,* strain CCAP 211/90
4	FM205862.1	*Chlorella colonials* strain UTEX 938
5	KF887350.1	*Chlorella variabilis* clone EdL_Cl2_3NB
6	KJ002639.1	*Chlorella thermophila* strain ITBB HTA1-65
7	KU948993.1	*Chlorella sorokiniana* isolate 34-2
8	KY229193.1	*Chlorella regularis* var. minima culture UTEX:1807
9	MK182466.1	*Chlorella salina* isolate MM0045
10	MT644459.1	*Chlorella pulchelloides* isolate 32-6
11	MT644460.1	*Chlorella volutis* isolate 32-7
12	MT644461.1	*Chlorella singularis* isolate 32-8
13	MT735073.1	*Chlorella* sp. MOW4
14	MT735075.1	*Chlorella* sp. MOW10 5.8S

Our strain sequences :
>NT3217_ITS Tetraselmis_10_cons
TCCGTAGGTGAACCTGCGGAAGGATCATTGAATCGATCAAACCCACTCCGTGAACTG
TTTTGTCTCCCTCTTGGGGCCGCTCGCGCGGCCGCTTGAGCGGCCAGGGATGCGTTC
CCTGGTCGGGCCTACCCCTGCGCCCCGGCGCGGGGGACCAGGTCGGCGCTCTTAAA
CAACCCACACCAAAACAACGTCTAAAGCTAAGTGCGCGTTGGTACTGCCGACCGCA
TCTAACCAAAGACAACTCTCAACAACGGATATCTTGGCTCTTACAACGATGAAGAAC
GCAGCGAAATGCGATACGTAGTGTGAATTGCAGAATTCCGTGAACCATCGAATCTTT
GAACGCATATTGCGCTCGAGGCCTCGGCCAAGAGCACGCCTGCCTCAGAGTCGGTTT
TCCCCCTCAACCCCCCTGTCCCAGGGCGGGCTGGACCTGGCAGTCTCAGGCCTCTTA
TGGCCTGGGTCCGCTGAAGTGCCGAGATTCAGCCAGGAACCCATCTATGGGCAAAC
ACTAGGTAGATAGCCTTCGGGTTATTCCTGTGTGTGTGTCTGCGGCCTGGCTGTGATC
CGCAGGAAAACAGAACCTTAACCATTCGACCTGAGTTCAGACGAGACTACCCGCCG
AACTTAAGCATATCAATAAGCGGAGGA

>NT3222 Chlorella _ITS_15_cons
TCCGTAGGTGAACCTGCGGAAGGATCATTGAATATGCAAACCACAACACGCACTCTT
TTATTTGTGTACCGACGTTAGGTCGAACCACTAACCCTGGTTTGGCCTACTAACCTAC
ACACACACCATTGACCAACATTTGATTAAACCAAACTCTGAAGTTTCAGCTGCTGTT
AATCGGCAGTTTTAACGAAAACAACTCTCAACAACGGATATCTTGGCTCTCGCAACG
ATGAAGAACGCAGCGAAATGCGATACGTAGTGTGAATTGCAGAATTCCGTGAACCA
TCGAATCTTTGAACGCATATTGCGCTCGACTCCTCGGAGAAGAGCATGTCTGCCTCA
GCGTCGGTTTACACCCTCACCCCTCTTCCTTAACAGGAAGCTTGTCGTGCTTGCTCAA
GCCGGCATCAGGGGTGGATCTGGCCCTCCCAATCGGACTCACCTCCGATTGGGTTGG
CTGAAGCACAGAGGCTTAAACTGGGACCCAATTCGGGCTCAACTGGATAGGTAGCA
ACACCCTCGGGTGCCTACACGAAGTTGTGTCTGAGGACCTGGTTAGGAGCCAAGCA
GGAAACGTGTCTCTGACGCGTATCTCTGTATTCGACCTGAGCTCAGGCAAGGCTACC
CGCTGAACTTAAGCATATCAATAAGCGGAGGA

Suupporting figure of un-cut gel, but the figure contains four strains each one containing four replicates of our strains. In this manuscript, we choose *Tetraselmis* lanes (5 to 8 we choose lanes 6 and 7), and another strain of *Chlorella* lanes (10 to 12 we choose lanes 10 and 11.

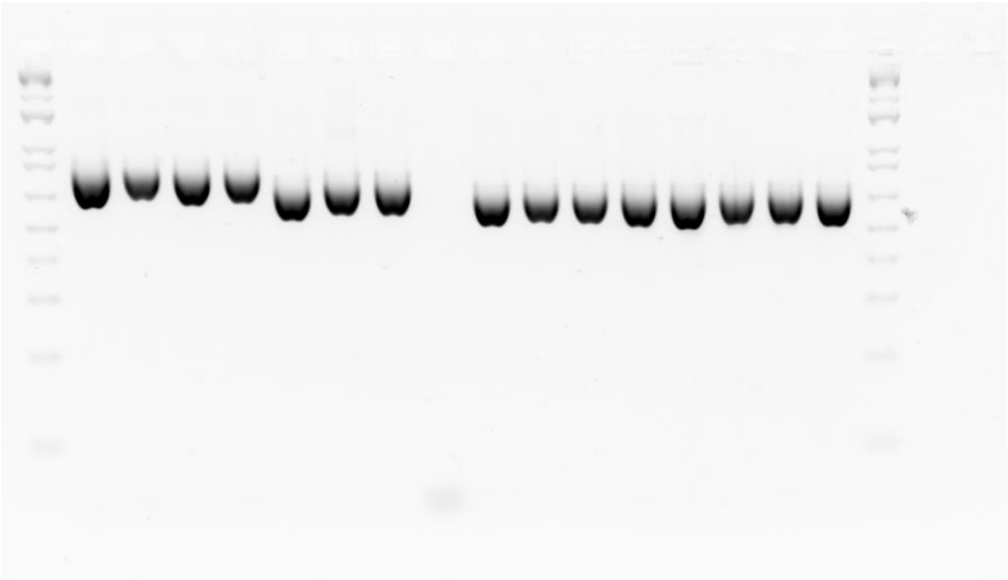

Micrograph of *Chlorella* under microscope :

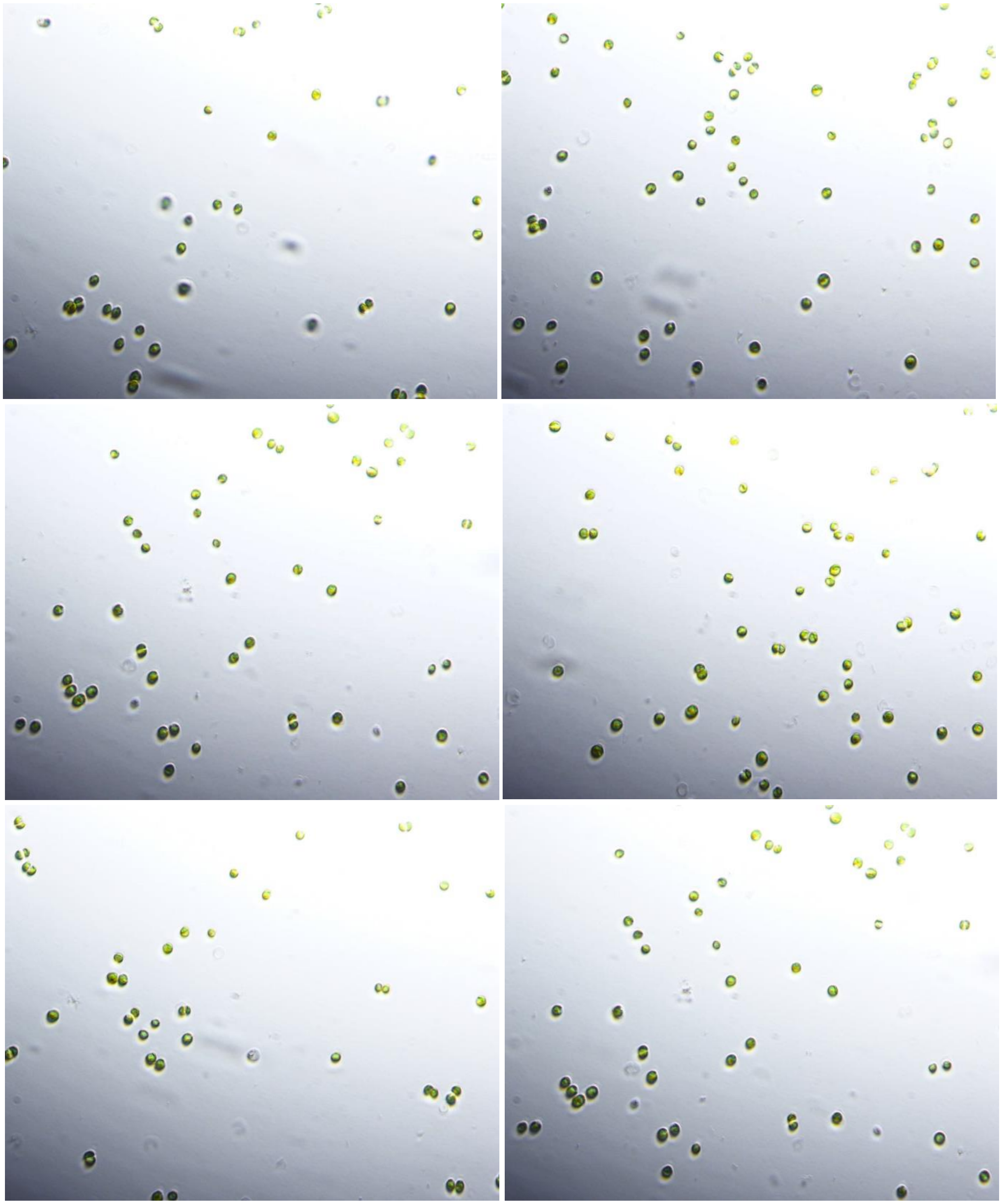

Micrograph of *Tetraselmis* under microscope :

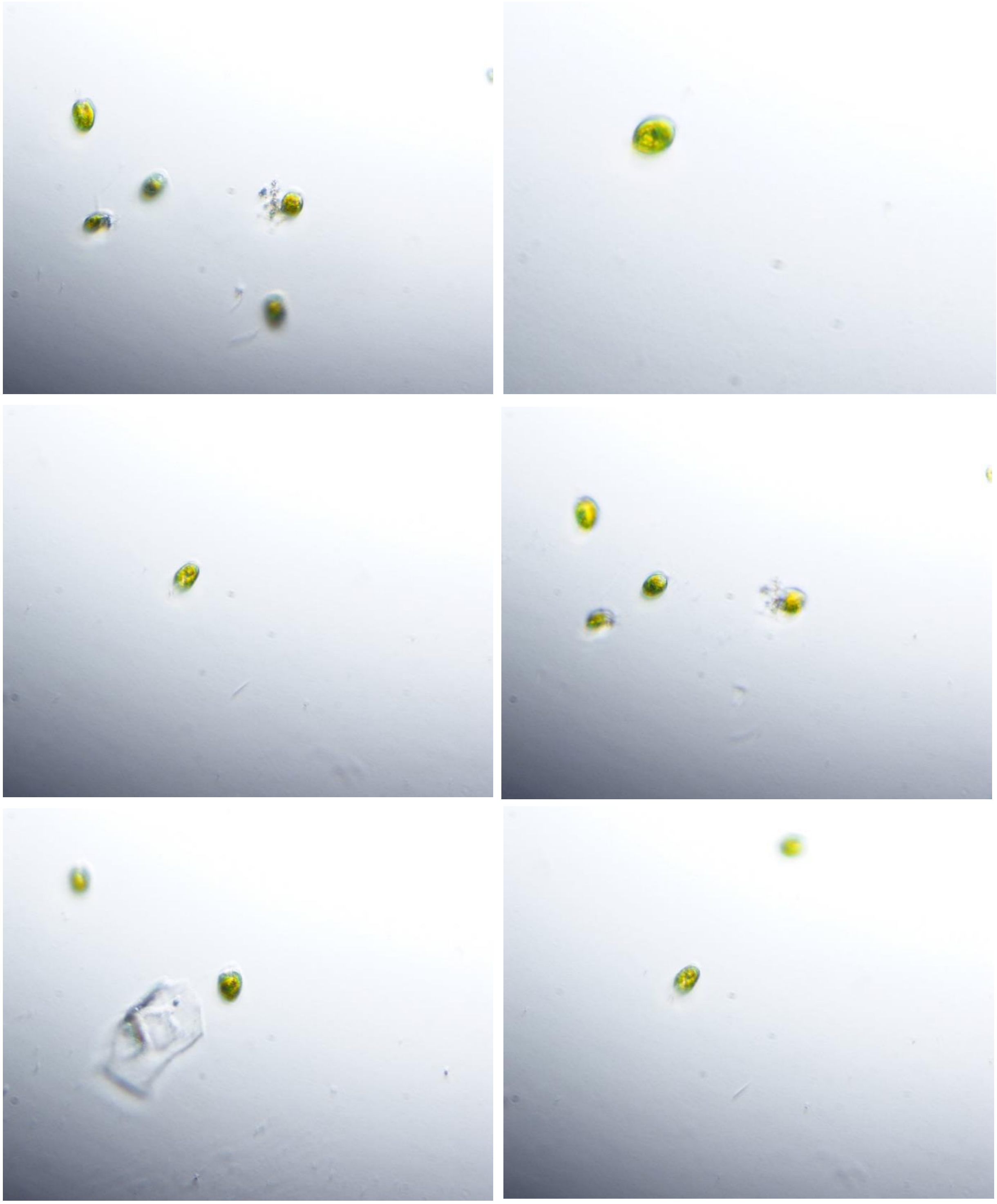

Growth conditions of *Chlorella* and *Tetraselmis*:

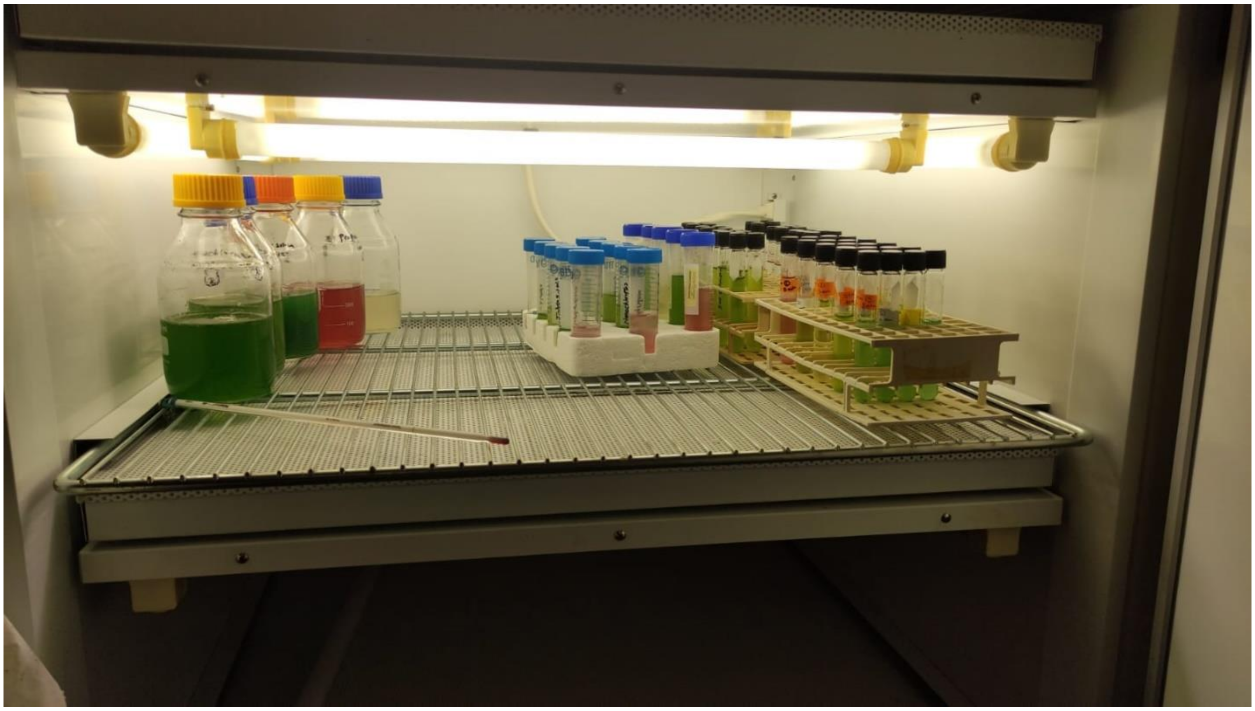

Supplement: Supplementary file 1 [file Data_Sheet_1.PDF]
