# Supplementary figures and images for: Applying an internal transcribed spacer as a single molecular marker to differentiate between Tetraselmis and Chlorella species
Source: Front Microbiol. 2023 Aug 23;14:1228869. doi: 10.3389/fmicb.2023.1228869 (PMC10482269; doi:10.3389/fmicb.2023.1228869)

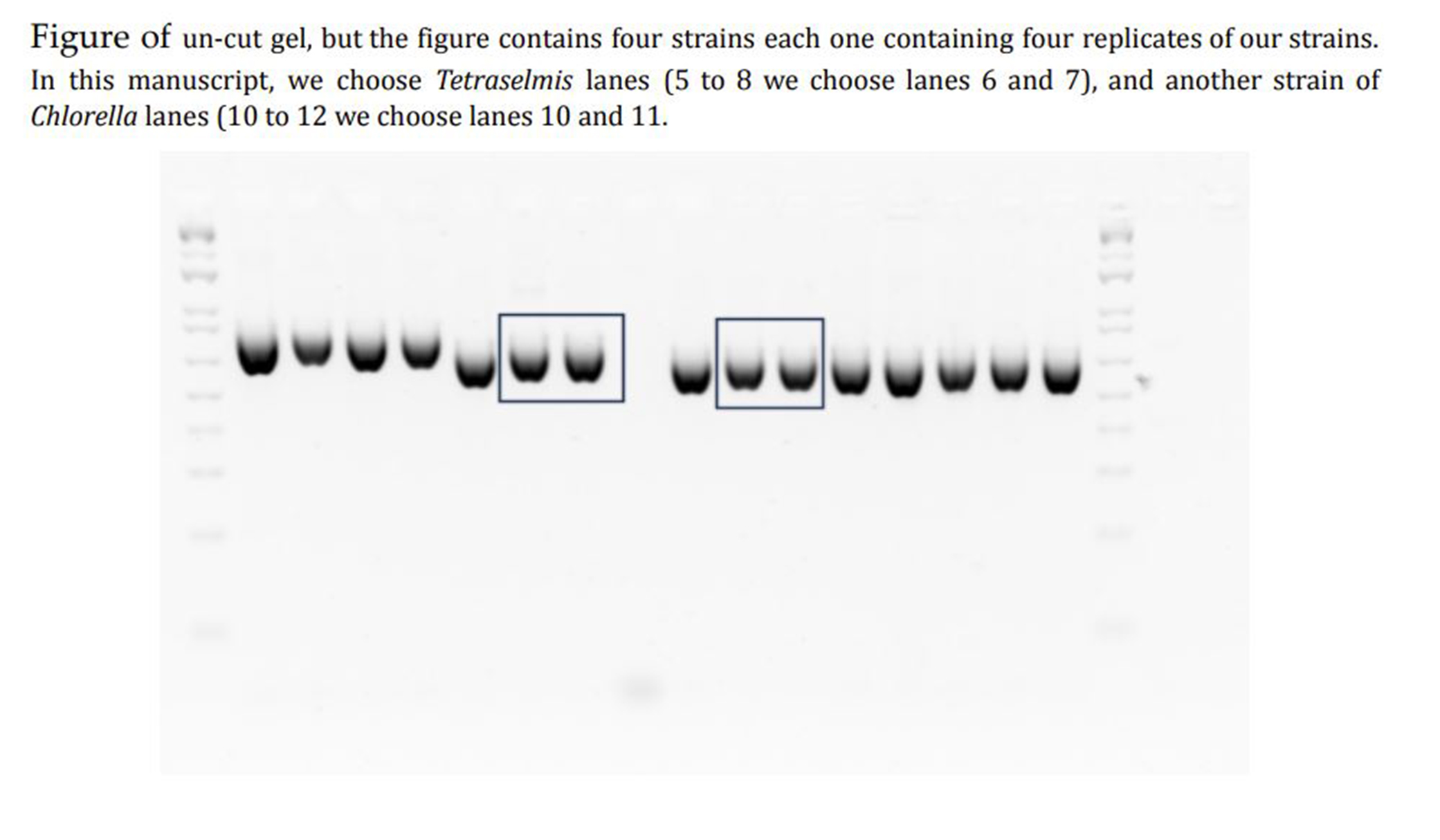

Supplement: Supplementary file 2 [file Image_1.JPEG]
